# Proteomics and Aging: Insights From the Osteoporotic Fractures in Men Study

**DOI:** 10.1093/geroni/igaf122.470

**Published:** 2025-12-31

**Authors:** Peggy Cawthon

## Abstract

Aging-related declines in muscle mass, frailty, and bone strength significantly impact health outcomes in older adults. This symposium presents cutting-edge research leveraging proteomics to uncover biological determinants of these age-related factors, using data from the Osteoporotic Fractures in Men (MrOS) Study. The first presentation examines proteomic determinants of muscle mass assessed using deuterated creatine dilution (D3Cr) and highlights key proteins, including novel targets like SVEP1 and brorin, that may play roles in muscle preservation. The second study explores frailty-related proteomic signatures, identifying 76 proteins associated with frailty, including markers linked to inflammation, immune response, and tissue repair. The third presentation focuses on proteomic predictors of bone loss, revealing site-specific skeletal protein associations and demonstrating that a multi-protein signature is a stronger predictor of bone loss than traditional biomarkers. Finally, the symposium concludes with an AI-driven analysis integrating proteomics and metabolomics to predict biological age, showcasing transformer-based models that outperform traditional machine learning approaches in identifying molecular markers of aging. Together, these studies advance our understanding of the molecular pathways underlying muscle loss, frailty, and bone deterioration in aging, while demonstrating the power of multi-omic and AI-based approaches in geroscience. This research paves the way for novel biomarkers and therapeutic targets to improve health outcomes in aging populations.

